# Efficacy and safety of AYUSH-64 as standalone or adjunct to standard care in COVID-19: a structured summary of protocol for a systematic review

**DOI:** 10.1186/s13643-022-01983-8

**Published:** 2022-05-24

**Authors:** Amit Kumar Rai, Azeem Ahmad, Pallavi Mundada, Krishna Kumar V, Babita Yadav, Shruti Khanduri, Bhogavalli Chandrasekhararao, Narayanam Srikanth

**Affiliations:** 1 Central Council for Research in Ayurvedic Sciences, Ministry of Ayush, Government of India, New Delhi, India; 2 National Ayurveda Research Institute for Panchakarma, Cheruthuruthi, Kerala India

**Keywords:** Ayurveda, AYUSH-64, Polyherbal, SARS-CoV-2, Pandemic

## Abstract

Registered with PROSPERO (CRD42021267844) dated 14.07.2021.

**Supplementary Information:**

The online version contains supplementary material available at 10.1186/s13643-022-01983-8.

## Background

Apart from vaccines, significant efforts have been made to develop prophylactic and therapeutic interventions against COVID-19. Lack of standard therapeutic options for the management of COVID-19 contributes to seriousness of this novel disease. The current strategy for exploring therapeutic interventions to manage this pandemic is broadly based on repurposing and repositioning of existing medications and recommending them for symptomatic support. In the early stage of COVID-19, the interventions that limit the progression of the disease and facilitate early recovery may play a significant role. AYUSH-64 is a polyherbal Ayurveda formulation developed by the CCRAS, Ministry of Ayush, Government of India. It has been found effective and safe in various infective febrile conditions like malaria, microfilaremia, chikungunya, and influenza [1–5]. AYUSH-64, was repurposed for the management of asymptomatic and mild to moderate COVID-19 based on the experimental and clinical outcomes indicating its potential benefits and safety in disease conditions like influenza-like illness. Government of India recommended the use of AYUSH-64 to manage the asymptomatic and mild COVID-19 cases on the basis of outcomes of clinical studies on AYUSH-64 in COVID-19 [6, 7]. In this context, this systematic review is planned to synthesize evidence related to the safety and efficacy of AYUSH-64 as standalone or adjunct to standard care in managing asymptomatic and mild to moderate COVID-19.

## Methods

The PRISMA-P (Preferred Reporting Items for Systematic Reviews and Meta-analyses- Protocol statement) guidelines have been followed for drafting this protocol. All Randomized Controlled trials that assess the efficacy and safety of AYUSH-64 for the management of COVID-19 as standalone or adjunct to conventional standard care will be considered for this systematic review. Comprehensive search will be done for the published studies as per the pre-designed search strategy in the electronic databases such as AYUSH Research Portal’s “National Repository on AYUSH COVID-19 Clinical and Other R&D Initiatives”, PubMed, Cochrane Central Register of Controlled Trials, DHARA, IndMED, COVID-19 Evidence Alerts from McMaster PLUSTM, Epistemonikos, TRIP database, Google Scholar, National Collaborating Centre for Methods and Tools database of COVID-19 studies, Clinical Trial Registry of India and WHO dashboard for clinical trials related to COVID-19 from inception till October 2021. Data selection and extraction for each study will be performed independently by two review authors, with disagreements resolved by discussion with third review author/consensus. Risk of bias assessment will be performed using the revised tool to assess the risk of bias in randomized trials (RoB 2). The results will be quantitatively synthesized (meta-analysis) using Review Manager 5.4. If meta-analysis will not be conducive due to substantial heterogeneity, we will summarize and explain the results of the included studies as the systematic qualitative synthesis.

## Discussion

To date, this will be the first systematic review on the Ayurveda intervention, AYUSH-64 that will synthesize the evidence on its efficacy and safety in the management of COVID-19. The pilot search undertaken before planning this systematic review resulted in 07 clinical studies on AYUSH-64 that evaluate its efficacy and safety in the management of COVID-19. These search results justify the initiation of this systematic review. This systematic review will help the policy makers for wider implementation of AYUSH-64 in the management of asymptomatic and mild to moderate COVID-19. The results of this systematic review will be published in an indexed open-access journal to ensure wider dissemination.

## Supplementary Information

**Additional file 1.** Full protocol of this systematic review.

**Additional file 2.** Example of search strategy (PubMed).

## Data Availability

Not applicable.
