# Supplementary material for: Efficacy and safety of AYUSH-64 as standalone or adjunct to standard care in COVID-19: a structured summary of protocol for a systematic review
Source: Syst Rev. 2022 May 24;11:103. doi: 10.1186/s13643-022-01983-8 (PMC9129058; doi:10.1186/s13643-022-01983-8)
Supplement: Supplementary file 1 — Additional file 1. Full protocol of this systematic review. [file 13643_2022_1983_MOESM1_ESM.docx]

**Title of the study:** Efficacy and Safety of AYUSH-64 as standalone or adjunct to standard care in COVID-19: A Systematic Review

**Registration:** Protocol Registered in PROSPERO on 14.07.2021(CRD42021267844)

**Support**: This study is not funded by any organization.

**INTRODUCTION**

**Rationale:** Corona virus disease (COVID-19) is an infectious disease caused by SARS-CoV-2 virus. World Health Organization (WHO) labeled it a Public Health Emergency of International Concern (PHEIC) on 30^th^ January, 2020 and a global pandemic on 11^th^ March, 2020. It is the third instance of a corona virus epidemic in the past two decades, after Severe Acute Respiratory Syndrome in 2003 and Middle East Respiratory Syndrome in 2012. The global scale of transmission, considerable number of deaths, infection and mortality of healthcare providers and higher risk of death in vulnerable population has been the major concern factors. The most common symptoms of COVID-19 are fever, fatigue, dry cough and dyspnea. Less common symptoms are nasal congestion, sore throat, diarrhoea, nausea, vomiting, loss of taste or smell, headache and dizziness. The most common complications of COVID-19 are severe pneumonia, acute respiratory distress syndrome, arrhythmias, acute cardiac injury, septic shock, venous thromboembolism and acute kidney injury.

Apart from vaccines, significant efforts have been made to develop prophylactic and therapeutic interventions against COVID-19. Lack of approved therapeutic options for the management of COVID-19 contributes to seriousness of this novel disease. The current strategy for exploring therapeutic interventions during pandemic is broadly based on repurposing and repositioning of existing medications and recommending them for symptomatic support. India has a well-established traditional medical system namely Ayurveda having scientific and holistic time-tested principles to prevent such epidemic diseases as well as their management in initial stage itself. In fact, Ayurveda is probably the oldest system of medicine in which infectious diseases and epidemics have been described in the context of *Aupasargika roga*, *Janpadodhvamsa*, *Agantuja vyadhi* etc. So, the prophylactic and therapeutic potential of Ayurveda need to be explored in the search for effective management of COVID-19 crisis. Given their traditional use has established safety, and experimental studies have demonstrated their immunomodulating, anti-inflammatory, anti-oxidant properties, and anti-viral activity, repurposing Ayurveda interventions for early stage COVID-19 is felt needed.

In the early stage of COVID-19, the interventions that limit the progression of the disease and facilitate early recovery may play a significant role. AYUSH‑64 is a polyherbal Ayurveda formulation developed by the CCRAS, Ministry of Ayush, Government of India. It has been found effective and safe in various infective febrile conditions like malaria, microfilaremia, chikungunya, and influenza. AYUSH‑64 was repurposed for the management of asymptomatic and mild to moderate COVID-19 based on the experimental and clinical outcomes indicating its potential benefits and safety in disease conditions like influenza like illness. Government of India recommended the use of AYUSH-64 to manage the asymptomatic and mild COVID-19 cases on the basis of outcomes of clinical studies on AYUSH-64 in COVID-19. Therefore to know whether AYUSH‑64 is effective and safe in the management of COVID-19 as standalone or adjunct to conventional standard care and to synthesize the level of evidence, a systematic review of the studies conducted to evaluate the efficacy and safety of this Ayurvedic formulation is of urgent need.

**Objectives:** To systematically summarize the available evidence of efficacy and safety of AYUSH-64 as standalone or adjunct to standard conventional care among asymptomatic, mild and moderate COVID-19 patients in facilitating early clinical recovery.

**METHODS**

**Eligibility criteria**

All Randomized Controlled trials that assess the efficacy and safety of AYUSH-64 for the management of COVID-19 as standalone or adjunct to conventional standard care will be included.

The following inclusion/exclusion criteria will be applied.

**i. Participants/population**

Patients of both sex and all age-groups, diagnosed with COVID-19 (asymptomatic / mild or moderate), with/without any pre-existing co-morbid conditions. Patients having severe COVID-19 will be excluded.

ii. **Intervention(s) and Comparator**

Studies on AYUSH-64 as standalone or adjunct to standard conventional care will be included. The studies in which AYUSH-64 was given along with any other Ayush intervention will be excluded. The comparator group will be conventional standard of care for asymptomatic and mild to moderate COVID-19 patients.

**iii. Outcomes**

**Primary outcomes:**

**Efficacy:** Mean duration to achieve ‘Clinical recovery’, Mean duration to achieve negative RT-PCR assay for COVID-19, Proportion of study participants with clinical recovery and negative RT-PCR assay for COVID-19, Change in the levels of pro-inflammatory markers, Change in the HRCT chest (CO-RADS score) and change in quality of life parameters.

**Safety:** Incidence of Adverse Events (AE)/ Adverse Drug Reaction (ADR) and change in haematological and biochemical parameters (complete blood count, liver function test, renal function test).

**Secondary outcomes:**

Average hospitalization time, conversion rate from mild to severe or critical stage of COVID-19 after treatment, and mortality.

**iv. Study design**

All Randomized Controlled Trials conducted after the emergence of the COVID-19 pandemic that assess the efficacy and safety of AYUSH-64 for the management of asymptomatic, mild or moderate COVID-19 patients as standalone or adjunct to conventional standard care. The published studies and preprints in English language, available in public domain will be searched.

Timings: From inception till October 2021.

Setting: There will be no restrictions by type of setting.

**Information sources**

The following information sources will be searched:

- 1. *Databases*: We will systematically search AYUSH Research Portal **“National Repository on AYUSH COVID-19 Clinical and Other R&D Initiatives”**, PubMed, Cochrane Central Register of Controlled Trials, DHARA, IndMED, COVID-19 Evidence Alerts from McMaster PLUS^TM^ , Epistemonikos, TRIP database, National Collaborating Centre for Methods and Tools database of COVID-19 studies and Google Scholar databases. The Clinical Trial Registry of India and WHO dashboard for clinical trials related to COVID-19 will also be screened. Study authors will be contacted if any information is required regarding the study. The search will be subsequently updated to include the most up to date data into the publication.
  2. Lists of references of eligible trials revealed by the database search will be screened for additional potentially relevant studies.
  3. Grey literature will be searched using Google Scholar. To do this, we will enter keywords and will continue search for relevant articles until the search does not reveal any article not captured by previous searches.

**Search strategy**

The search strategy is based on the following three concepts joined by the Boolean operator “AND”: (1) Intervention (“AYUSH-64” OR Ayurveda* OR “Ayurvedic therapy” OR “Ayurvedic treatment” OR “Ayurveda intervention” OR “Ayurvedic management” OR Polyherbal formulation); (2) Disease (COVID OR COVID-19 OR “Corona Virus” OR “Corona Virus Disease” OR “2019 novel coronavirus infection” OR “2019-nCoV disease” OR “SARS-CoV-2” OR Pandemic OR “Severe acute respiratory syndrome”); (3) Study design (“Clinical trials” OR “Clinical trial” OR “RCT” OR “Randomized controlled trial” OR “Randomized controlled study”)

A draft of search strategy to be used for PubMed is enclosed.

**Study records**

**Data management**

The collected data from all the available sources will be kept secure. A duplicate copy will also be created and kept in an external data storage device; it will be accessible only to the authors of the review.

**Selection process**

Two authors (KK and PM) will independently screen all the results. If there will be any disagreement between these two authors, it will be resolved by a discussion with the third author (AKR). Where there will be still any doubt, the full article will be acquired for further inspection. Once the full article will be obtained, two authors (KK and PM) will decide whether the study met the review criteria.

**Data collection process**

Two reviewers (AKR and AA) will assess the eligibility of the searched studies independently using the inclusion and exclusion criteria. We will make a pre-designed format to extract data from the included studies for further data analysis. The following study items will be extracted: authorship, publication-related information, methodology, participants, interventions, comparators, and outcomes (efficacy and safety). The investigators of eligible trials will be contacted through email to seek clarity of desired information if required.

**Data items**

**Participants:** Patients of both sex and all age-groups diagnosed with asymptomatic or mild-moderate COVID-19, with/without any pre-existing co-morbid conditions. We will also collect details such as patient characteristics (average age, gender, stage of the disease, etc.), study design, and sample size.

**Interventions:** Dose and duration of AYUSH-64 as standalone or adjunct to standard conventional care.

**Comparators/ Control:** Standard conventional care as per existing guidelines issued by Government authorities.

**Outcomes and prioritization**

**Primary outcomes:**

**Efficacy:** Mean duration to achieve ‘Clinical recovery’, Mean duration to achieve negative RT-PCR assay for COVID-19, Proportion of study participants with clinical recovery and negative RT-PCR assay for COVID-19, Change in the levels of pro-inflammatory markers, Change in the HRCT chest (CO-RADS score) and change in quality of life parameters.

**Safety:** Incidence of Adverse Events (AE)/ Adverse Drug Reaction (ADR) and change in haematological and biochemical parameters (complete blood count, liver function test, renal function test).

**Secondary outcomes:**

Average hospitalization time, conversion rate from mild to severe or critical stage of COVID-19 after treatment, and mortality.

**Risk of bias in individual studies**

The methodological quality of included Randomized Controlled Trials (RCTs) will be assessed by using the revised tool to assess the risk of bias in randomized trials (RoB 2) which is available online. Five domains viz. randomization Process, deviations from intended interventions, missing outcome data, measurement of the outcome, and selection of the reported results will be assessed with the help of pre-defined algorithms and will be represented in Traffic light plot and Weighted Summary Plot.

**Data synthesis**

The study data will be quantitatively synthesized (meta-analysis) if sufficient studies are clinically and methodologically similar, with statistically similar treatment effects. For dichotomous data, odds ratio will be used, whereas the mean difference will be used for continuous outcomes to measure the treatment effect with 95% confidence intervals. Heterogeneity among trials will be assessed by applying the Chi-square test and using the I^2^ statistic. A random-effects model will be used. Meta-analyses will be completed using Review Manager 5.4.

If meta-analysis will not be conducive due to substantial heterogeneity, we will summarize and explain the results of the included studies as the systematic qualitative synthesis. If significant heterogeneity exists, we will perform subgroup analyses to explore the possible causes. Pre-planned categories for subgroup analyses:

- Patient demographics (e.g. age, pre-existing co-morbidities like cardiovascular disease, diabetes mellitus, hypertension, and kidney function abnormalities, etc.)
- Study characteristics (e.g. study design, sample size, duration of follow-up, study quality, etc.)
- Stage of disease condition: Asymptomatic or mild to moderate cases of COVID-19

**Meta-bias(es)**

Publication bias and selective reporting of outcomes within the included studies will also be assessed.

**Confidence in cumulative evidence**

The Grading of Recommendations Assessment, Development, and Evaluation (GRADE) framework will be used to evaluate the strength of the cumulative evidence. The assessment will include all the studies included in the review.

**Ethics and dissemination**

This review does not require formal ethical assessment and approval, as no confidential participant data will be included. The protocol for this systematic review has been registered with PROSPERO, the International Prospective Register of Systematic Reviews.The results of this systematic review will be reported as per the Preferred Reporting Items for Systematic Reviews and Meta-Analyses (PRISMA) guidelines and published in an indexed open-access journal to ensure wider dissemination.
