# Supplementary material for: Efficacy and safety of AYUSH-64 as standalone or adjunct to standard care in COVID-19: a structured summary of protocol for a systematic review
Source: Syst Rev. 2022 May 24;11:103. doi: 10.1186/s13643-022-01983-8 (PMC9129058; doi:10.1186/s13643-022-01983-8)
Supplement: Supplementary file 2 — Additional file 2. Example of search strategy (PubMed). [file 13643_2022_1983_MOESM2_ESM.docx]

**Search Strategy**

**PubMed database**

1. (“AYUSH-64” OR Ayurveda* OR “Ayurvedic therapy” OR “Ayurvedic treatment” OR “Ayurveda intervention” OR “Ayurvedic management” OR Polyherbal formulation)
2. (COVID OR COVID-19 OR “Corona Virus” OR “Corona Virus Disease” OR “2019 novel coronavirus infection” OR “2019-nCoV disease” OR “SARS-CoV-2” OR Pandemic OR “Severe acute respiratory syndrome”)
3. (“Clinical trials” OR “Clinical trial” OR “RCT” OR “Randomized controlled trial” OR “Randomized controlled study”)
4. (1 AND 2 AND 3)
